# Nonfunctioning pituitary macroadenoma: a case report from the patient perspective

**DOI:** 10.1186/s12998-016-0093-z

**Published:** 2016-04-11

**Authors:** Craig A. Bauman, James D. Milligan, Tammy Labreche, John J. Riva

**Affiliations:** Department of Family Medicine, McMaster University, Hamilton, ON Canada; The Centre for Family Medicine Family Health Team, 25 Joseph Street, Kitchener, ON Canada N2G 4X6; School of Optometry and Vision Science, University of Waterloo, Waterloo, ON Canada; Department of Clinical Epidemiology & Biostatistics, McMaster University, Hamilton, ON Canada

**Keywords:** Pituitary, Adenoma, Macroadenoma, Nonfunctioning, Middle-aged, Athletic, Performance

## Abstract

**Background:**

Nonfunctioning pituitary macroadenoma (NFPA) is a tumour of the endocrine system that is virtually always benign and can be difficult to detect. This case report is presented from the patient’s perspective to highlight experiences that led to the eventual diagnosis of this condition.

**Case presentation:**

A 48 year-old male experienced prolonged and unexplained reduced athletic performance worsening over five years. The patient reported decreased libido, which initiated a testosterone blood test. This confirmed reduced testosterone levels and resulted in an endocrinology referral. A subsequent dynamic contrast MRI of the pituitary region revealed a mass. The most frequent symptoms of NFPA are visual field defects, headaches and features of hypopituitarism (includes fatigue, dizziness, dry skin, irregular periods in women and sexual dysfunction in men).

**Conclusion:**

Clinicians should consider this differential diagnosis in middle-aged athletes with diminished athletic performance from an unknown cause, test visual fields and inquire if symptoms of headaches or hypopituitarism are present.

## Background

Chiropractors are commonly utilized by middle-aged athletes to determine sources of reduced athletic performance [1]. Such clinicians are also often consulted for headache treatment [2]. For this reason chiropractors need to be aware of the potential diagnosis of nonfunctioning pituitary macroadenoma.

**Fig. 1:**
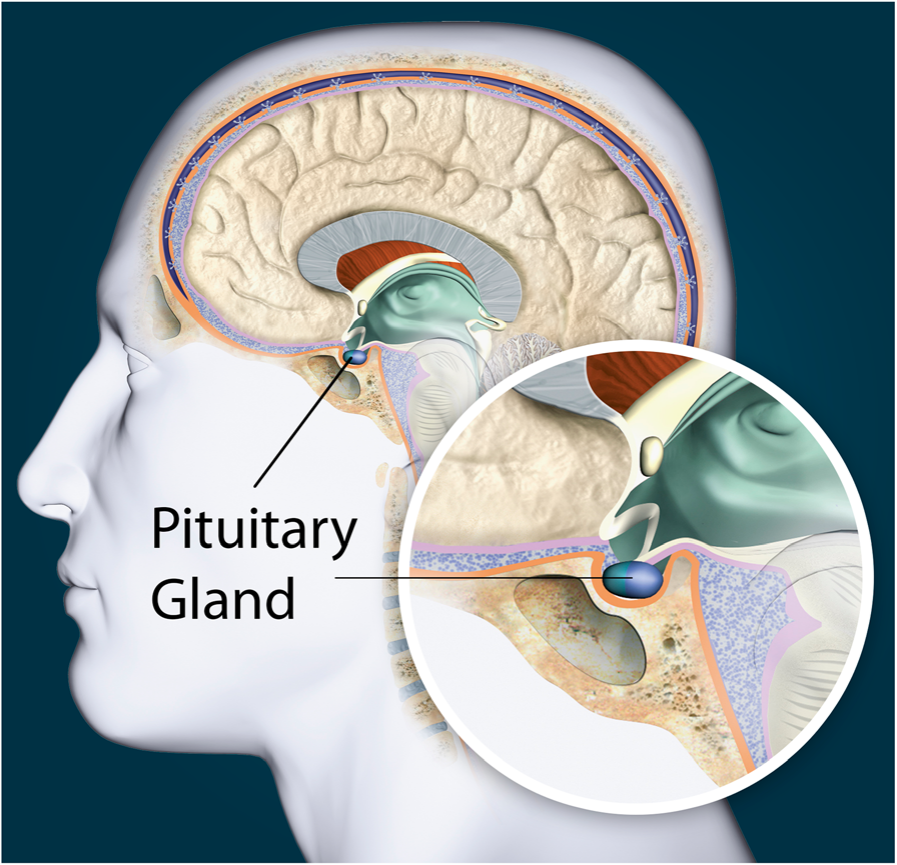
Position of the Pituitary Gland in the Head. With permission from the Pituitary Society and licenced from Thinkstock by Getty Images

**Fig. 2:**
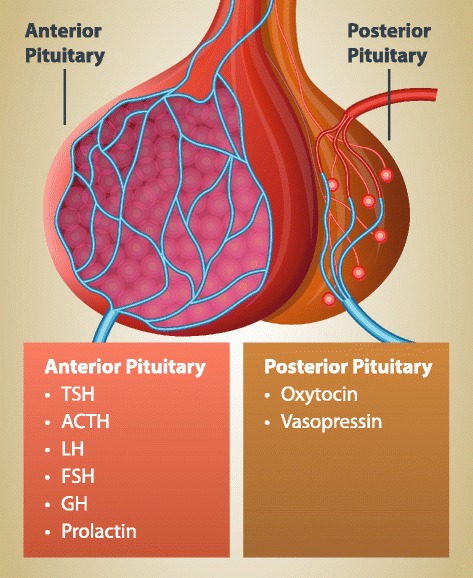
Hormones of the Anterior and Posterior Pituitary Gland. TSH is thyroid stimulating hormone, ACTH is adrenocorticotropic hormone, LH is luteinizing hormone, FSH is follicle-stimulating hormone and GH is growth hormone. With permission from the Pituitary Society and licenced from Thinkstock by Getty Images

The pituitary is an endocrine (hormone-producing) gland that is located immediately below the base of the brain (Figs. 1 and 2) [3]. It approximates the size of a pea and is regulated by the hypothalamus [3]. It produces hormones that impact numerous parts of the body and stimulates all the other endocrine glands to produce their own hormones [3]. As a result of its complex tasks, it is frequently referred to as the ‘master gland’ [3].

Nonfunctioning pituitary macroadenoma (NFPA) is a nearly always benign tumour of the endocrine system [3, 4]. NFPA is the most common adenoma in the pituitary gland, accounting for 25–33 % of these tumours [5, 6]. At the time of diagnosis, most NFPAs are macroadenomas (greater than 1 cm in diameter) [5, 6]. The prevalence of NFPA has been estimated at 22 cases per 100 000 in cross-sectional studies [4]. They frequently present in those greater than 50 years of age [5], and the progression can vary widely, with some growing slowly and others becoming rapidly invasive [5].

The gold standard method of management of NFPA is surgical resection of the tumour via endoscopy using a transsphenoidal approach through the nasal cavity [4]. Prognosis for treated patients is generally very good [4]. Although NFPA is benign in nature, patients often need individualized management, lifelong diagnostic imaging and endocrinological monitoring [7]. This paper describes a specific case presentation around diminished athletic performance to highlight typical features related to NFPA from the patient perspective.

## Case Presentation

### History

I am a Canadian Doctor of Chiropractic and chiropractic clinician who is sharing their experience with NFPA. Fortunately, through fate or luck, circumstances led to successful management. My story starts as an age 43 male, five years prior to diagnosis. After an afternoon nap, I arose from bed quickly and had a brief intense pain sensed in the centre of my head. For 1 month afterwards, I had a mild sharp pain in the centre of my head for the first 200 m of a recreational run. I attended my family physician for this complaint and had a neurological examination, which was normal, and I was advised to come back if the pain returned. In hindsight, I felt this head pain was related to the NFPA; however this was not the typical type of headache presentation. Over the next 5 years I noticed a slow progressive reduction in strength that corresponded to a 30 % decrease in my weight training ability. Also, I noticed a weight gain of 5–10 lb that was mostly abdominal which I initially thought was attributed to “getting older”. When I ran a competitive 5-km race which resulted in me being 2 min and 12 s slower than the previous year despite increased training, this prompted me to return to my family physician.

Bloodwork was ordered. A mild anemia (normochromic normocytic) was detected, with hemoglobin of 117 g/L (normal 135–175 g/L), hematocrit of 0.36 L/L (normal 0.40–0.50 L/L), and red blood cell count of 3.83 x E12/L (normal 4.50–6.00 x E12/L). Investigations to determine the etiology of the anemia included a chest x-ray, colonoscopy, gastroscopy and abdominal ultrasound, all of which were normal. During this time I reported to my family physician I was losing hair on my legs, my libido was very low along with having acquired cold sensitivity, hot flashes, anxiety and some noticeable hair growth on the scalp where I had been losing hair. By chance, during the period of when various diagnostic tests were occurring, I attended a family medicine continuing education conference where an endocrinologist spoke of the connection between low testosterone and a pituitary tumour. I later discussed this possibility with my family physician and a testosterone blood test was ordered.

### Physical examination

The results of the testosterone tests showed testosterone was nearly absent at 0.5 nmol/L (normal 8.4–28.8 nmol/L) and bioavailable testosterone was 0.1 nmol/L (normal is 3.6–11.2 nmol/L). I was referred to an endocrinologist. A dynamic contrast MRI of the pituitary region (specifically the sella turcica) was ordered. A large 2.5 cm diameter mass was discovered in my pituitary region. A subsequent referral was made to a pituitary neurosurgical team and a series of tests and consults were booked (further bloodwork, neuro-opthalmology and endocrinology appointments). My bloodwork identified reduced pituitary hormone levels, which suggested the tumour was nonfunctioning. The luteinizing hormone (LH) at 0.8 IU/L (normal male 1.7–8.6 IU/L) and follicle stimulating hormone (FSH) at 1.1 IU/L (normal male 1.5–12.4 IU/L) were also reduced. Diminished levels of these hormones were causing my testosterone to be low, which compromised hematopoiesis (i.e. the production of all types of blood cells) and resulted in the anemia [8]. Prolactin was elevated to 51 ug/L (normal male 4–15 ug/L) due to the tumour stalk effect (i.e. mass effect from the tumour on the pituitary infundibulum) [7, 9]. Thyroid function was also compromised by the NFPA. My Free T4 was 11 pmol/L (normal 12–22 pmol/L) and Free T3 was 2.8 pmol/L (normal 3.1–6.8 pmol/L). I was prescribed testosterone gel and levothyroxine medication for replacement.

While frequently affected in NFPA, the neuro-ophthamologist concluded my visual fields were normal. A consult with an otolaryngologist (ENT) surgeon was required to evaluate the size of my nasal cavity in preparation for the surgical endoscopy procedure. In surgery, the endoscope is passed through the nasal cavity to make an opening in the sphenoid sinus, to access the pituitary region allowing the neurosurgeon to remove the tumour. This is called the transsphenoidal approach and its advantages are being less invasive and no visible scaring following neurosurgery [9].

### Imaging

The neurosurgeon and the neuroradiologist reviewed my imaging (the previous dynamic contrast MRI and a recent CT) and had a high degree of suspicion of a NFPA. Only after surgery and pathological sectioning of the tumour can a confirmatory diagnosis be made.

### Intervention

Prior to surgery, I received meningococcal B immunizations as ENT surgery has an eleven-fold and neurosurgery has a seven-fold, increased risk of meningitis in the first 10 days following surgery [10]. With a MRI just before surgery, adhesive stickers known as fiducial markers were attached to my head. This assists the neuro-navigation of the neurosurgeon to help orient themselves relative to the tumour position in my head during the surgery. In the operating room, my head was placed in a form-fitting cradle for stabilization. Later, when under anesthetic, a series of pins were screwed into my skull to further stabilize it, as the surgeon has to be very precise during the procedure. The entire surgical procedure typically takes up to 5 h.

## Conclusions

The surgery was completed without incident and I was returned to the recovery room after approximately 4 h. I had a subsequent follow up with my care team one month after surgery, where the pathological sectioning confirmed a NFPA. This was a tremendous relief as other, more sinister tumours were a possibility. Radiation therapy was considered but not deemed required. Future ongoing screening recommendations for recurrence includes annual MRI monitoring.

## Discussion

### Clinical presentation

Clinically, NFPA comprise about 80 % of all pituitary macroadenomas [5]. It is likely there are many in the population with undetected macroadenomas [11]. Imaging of the head is now commonly implemented as part of the workup of many unrelated medical cases [5] and as a result, pituitary lesions are often detected incidentally [5]. Nonfunctioning categorizes the tumour cells as not secreting hormones versus functioning adenomas. This can be advantageous; but also can make discovery difficult as there is absence of a readily identifiable syndrome of pituitary hormone hypersecretion often leading to growth of these tumours for many years before discovery [9]. Many cases presenting with NFPA have at least some pituitary insufficiency [7]. The majority of patients who have NFPA will seek medical attention because of a mass effect (i.e. the growing tumour applying pressure to tissues) from the macroadenoma [9]. The main complaints or symptoms associated with mass effect are visual field defects with or without decreased visual acuity, unspecified headache and effects of hypopituitarism (Table 1) [3]. As a result, many macroadenomas are often uncovered at the optometrist [9].

**Table 1:** Symptoms and Signs of Pituitary Hormone Deficiency. With permission from the Pituitary Society

Pituitary Hormone	Target Organs	Effect of Deficiency
ACTH	Adrenal glands: cortisol and DHEA	Fatigue, low sodium in blood, weight loss, skin pallor
TSH	Thyroid gland: thyroid hormone	Fatigue, weight gain, dry skin, sensitivity to cold, constipation
LH and FSH in Women	Ovaries: estrogen, progesterone; ovulation	Loss of periods, loss of sex drive, infertility
LH and FSH in Men	Testes: testosterone, sperm production	Loss of sex drive, erectile dysfunction, impotence, infertility
GH in Children & Adolescents	Bone, muscle, fat	Lack of growth (height); increased body fat, failure to achieve normal peak bone mass
GH in Adults	Whole body	Poor quality of life, increased body fat, decreased muscle and bone mass
PRL	Breast	Inability to breast feed
Oxytocin	Breast, Uterus	Complete deficiency could make breast feeding difficult
Antidiuretic hormone (vasopressin)	Kidney	Frequent urination (day & night), dilute urine, excessive thirst

Visual field defects exist in 60–70 % of patients with NFPA at the time of suspicion of the diagnosis, which results from pressure on the optic chiasm by the tumour [7, 9]. Patients are usually unaware of the deficiency, which only becomes apparent during formal visual field testing [9]. Compression of the chiasm can result in a symmetrical bi-temporal hemianopia (i.e. loss of the outer field of vision in both eyes), but more commonly there is an asymmetrical bi-temporal hemianopia or even a unilateral temporal field defect [5, 7]. The chiropractic clinician could test visual fields if NFPA is suspected using the confrontation visual field test. Headache, often localized to the brow or periorbital region, is present in 40–60 % of all patients and is generated by increased intracranial pressure and/or stretching of the dura mater [7, 12]. The neurosurgeon stated that NFPA, along with some other pituitary tumours, are increasingly detected in middle-aged athletes with reduced athletic performance and signs of hypopituitarism. Inquiry as to the presence of these symptoms should be made in the patient history. As chiropractors are consulted by mature athletes, NFPA should remain in their differential diagnosis when such signs are present and a referral made to their physician with a note.

Differential diagnoses of NFPA includes craniopharyngiomas, germinomas, metastatic tumours and vascular aneurysms [13]. Differential diagnoses for NFPA visual field defects includes compressive lesions (eg, craniopharyngiomas), ischemic lesions (eg, pituitary apoplexy), inflammatory lesions (eg, multiple sclerosis), and toxic lesions (eg, pheniprazine) [12]. Differential diagnoses of NFPA headaches includes cluster headaches, migraine variants and trigeminal neuralgia [14]. Fatigue could be caused by infection, anemia, endocrinopathies (eg, diabetes and hypothyroidism), sleep disturbances (eg, sleep apnea), medication side-effects, adrenal insufficiency (usually other symptoms and signs) and malignancies (rare) [15]. Overtraining syndrome in athletes can also lead to fatigue [16].

Pituitary tumours can be classified by size or by function [5]. The best current diagnostic imaging method for evaluating pituitary adenomas is MRI [7]. Size, as determined by MRI, less than 1 cm in diameter is considered a microadenoma and those tumours greater than 1 cm in diameter are considered a macroadenoma [5, 11]. Function is described by the detectable increase of a pituitary hormone through blood tests [5]. Therefore, if no hormones were manufactured, the tumour is defined nonfunctioning. These tumours may also be classified according to immunohistochemistry [11].

NFPAs that touch the optic apparatus, without visual dysfunction, may be followed with close ophthalmological and radiographic monitoring, pending tumour and imaging characteristics [17]. Surgery should be contemplated for those patients with concerning tumour growth, loss of endocrinological function, a lesion close to the optic chiasm, a desire to become pregnant, or unremitting unspecified headaches [18].

### Treatment

The majority of patients with a large NFPA should have pituitary surgery [5, 6, 9]. Surgical morbidity and cure rate have been found to be highly reliant on the skill of the pituitary surgeon [7, 9, 19]. The favoured surgery is a transsphenoidal approach, due to less associated morbidity and mortality [9]. The surgical management of macroadenomas (greater than 1 cm) is precipitated by the greater probability that these tumours will increase in size over time leading to resultant mass effects. The initial goals of post surgical therapy are to mitigate any future mass effect and to return normal endocrine function [9].

Because there is a known correlation between the severity of visual loss before surgery and persisting visual field defects after treatment, the wait time for surgery is typically expedited [7]. Besides the improvement of visual function, full cessation of headaches is likely to occur after surgery for NFPA [7]. During post surgical follow-up, careful assessment and replacement of pituitary deficiencies is implemented [7]. It is possible that future advancements in the field of neurosurgery, such as endoscopic techniques utilizing combination with neuronavigation, will further improve surgical outcomes and improve the long-term prognosis [7].

NFPA treatment can involve single or combinations of surgical intervention, radiotherapy or pharmacological treatment [5]. It is essential that all options for therapy are discussed and inclusion of surgical, endocrine and oncology providers is often provided in a multidisciplinary team setting [5]. For those adenomas less than 1 cm in diameter and restricted to the sella, a strategy of watchful waiting and serial MRI scans is often employed [5]. It is necessary to have ruled out any secondary hormonal deficiencies to optimize quality of life [5].

Assuming hormonal normality, then close monitoring may be all that is needed [5]. With no hormone irregularity present, follow up in this instance relies upon serial screening [5]. For those adenomas larger than 1 cm (the majority), after excluding hormone deficiencies, it is common to proceed to surgical management [5]. These macroadenomas more commonly exert pressure effects [5].

Pharmacological treatment of the NFPA tumour itself is typically unsuccessful [5, 9]. Medical management is frequently based around regular follow-up, monitoring of visual problems and secondary hormone deficiencies [5]. Periodically, tissue regrowth develops post surgically and, although a repeat surgical intervention can be pursued, adjunctive radiotherapy also remains a useful option [5]. Indeed, radiation therapy may be proposed if the patient is not suited for surgery or if complete tumour removal is not achievable [5, 20, 21]. Radiosurgery for NFPAs is quite effective, with usually around a 90 % success rate for tumour control [20, 21].

The greatest risk of transsphenoidal neurosurgery is a postoperative cerebrospinal fluid leak [4]. When this occurs, a clear fluid drips out of the nose quite steadily when leaning forward. Symptoms of a leak may also include a headache, however headache can also be expected after the neurosurgical procedure itself. Post-surgery, rest and avoidance of straining activities (valsalva) is required with an anticipated return to work in approximately 2 months.

All patients that require hydrocortisone replacement should carry a steroid card (e.g. instructions in case of an emergency) and participate in education about sick day rules (sickness stress requires increased cortisol which can no longer be produced) [5]. These sick day rules will be advised by a physician and frequently involves doubling the usual hydrocortisone dose for 1 to 3 days [22]. An adrenal crisis (acute cortisol insufficiency) can ensue during times of illness which can be life threatening and must be managed promptly [23]. Symptoms include unusual tiredness and weakness, dizziness when standing up, nausea, vomiting, diarrhea, loss of appetite, stomach ache and joint aches and pains [22].

### Prognosis and adverse events

In NFPA, no single conclusive predictive factor has been identified that is associated with recurrence [24]. NFPAs, with or without perceptible residual tumour, need stratification of treatment and radiological/endocrinological follow-up strategies [6]. The tumour growth-free survival rates at 5 years are 85 % with intrasellar remnant and 49 % with extrasellar remnant and at 10 years are 58 % with intrasellar remnant and 23 % with extrasellar remnant [6]. Fortunately, 54 % of residual tumours will not regrow again after surgery [6, 9]. Therefore, it is advised that MRI should be repeated annually in these patients [6]. Reoperation or radiotherapy should be contemplated only when the tumoural regrowth is confirmed, except where the adenomatous residue is voluminous and close to the optic nerves/chiasm [6]. Whether NFPAs are associated with an increased mortality or reduced lifespan is still unknown [8].

Adverse events from a transsphenoidal approach were linked with 1 % mortality and 5 % important complications (e.g. cerebrospinal fluid leakage, fistula, meningitis, persistent diabetes insipidus or new visual field defect) [4]. Olfaction may be impacted by this surgical technique [25, 26]. Of patients with visual field defects prior to surgery, 78 % had recovery in their visual field defects [4]. Conversely, gains in pituitary function occurred in less than a third of the patients [4]. Complete removal of the lesions, as evaluated by the operating surgeons, occurs in approximately 20 % of the cases [4].

### Limitations

A key limitation of this paper may be the inherent/unintentional bias that the principal author may bring to the report since it’s from the patient-perspective. This case report may have biased observations in how the principal author recounted the details.

### Case conclusion

One month after surgery, I was able to stop taking hydrocortisone as my pituitary returned to producing sufficient hormone. I was off work a total of 6 weeks. Six months post surgery, I felt I was in excellent health, had returned to running and strength training and my body weight had returned to the level it was 5 years prior. The 6 month follow up dynamic contrast MRI showed no definite tumour residual. Ongoing yearly MRIs will occur to monitor for regrowth. I will continue on testosterone and levothyroxine medication indefinitely and levels monitored. Truly, I am delighted with the outcome and decided to share the learning experience through a case report for other clinicians as a patient may describe these slowly progressive symptoms during a consultation.

### Take home points

- Nonfunctioning pituitary macroadenoma can be difficult to detect
- Vision changes, headaches and symptoms of hormone irregularity offer clues to the tumour's presence
- Unexplained reduced performance in middle-aged athletes may be an indicator
- Post-surgery, yearly imaging may be required to monitor for tumour recurrence
- Life-long hormone replacement therapy may be required

## Consent

Written informed consent was obtained from the patient for publication of this case report. A copy of the written consent is available for review by the Editor-in-Chief of this journal.
